# Influence of Different Environments and Temperatures on the Photo-Oxidation Behaviour of the Polypropylene

**DOI:** 10.3390/polym15010074

**Published:** 2022-12-24

**Authors:** Francesco Paolo La Mantia, Marilena Baiamonte, Stefania Santangelo, Roberto Scaffaro, Maria Chiara Mistretta

**Affiliations:** 1 Department of Engineering, Università di Palermo, Viale delle Scienze, 90128 Palermo, Italy; 2 INSTM, Italian Consortium for Materials Science and Technology, Via Giusti 9, 50125 Firenze, Italy

**Keywords:** polymers, photo-oxidation, environments, sea water, oxygen solubility

## Abstract

The photo-oxidation of polypropylene at two different temperatures and in three different environments—air, distilled water and sea water—has been followed as a function of the irradiation time. The photo-oxidation kinetic is dramatically dependent on the amount of oxygen available for the oxidation reactions and on the temperature. While the photo-oxidation is very fast in air, the degradation is much slower in the two aqueous media. The degradation in sea water is slightly slower than in distilled water. In all cases, the degradation kinetic increases remarkably with the temperature. This behavior has been attributed to the lower oxygen availability for the oxidation reactions of the polymers. The light difference of the degradation kinetic between the two aqueous media depends on the small difference of the oxygen concentration at the test temperatures of 40 and 70 °C. At the latter temperature, the difference between the degradation kinetic in distilled water and sea water is still less important because increasing the temperature decreases the solubility of the oxygen, and it tends to became very similar in both samples of water.

## 1. Introduction

The photo-oxidation behavior of polymers depends on the chemical composition, molecular structure and morphology of the polymers, but also on the physical conditions of the environment, i.e. UV irradiation, moisture, presence of oxygen and temperature. Oxygen and temperature play a significant role in the photo-oxidation kinetic, and the photo-oxidation itself takes place only in the presence of oxygen. This feature is significant if photo-oxidation occurs when the polymers are in water or marine water [1,2,3]. The degradation of polymers in sea water is of remarkable importance due to the dramatic consequences that the fragmentation of plastics into microplastics has on the marine environment [4,5,6,7]. The seawater plays some protective role in the photo-oxidation of polymers, and the lower photo-oxidation kinetic in water, and marine water in particular, compared to photo-oxidation in the air has been attributed to the low oxygen concentration in marine water [8,9,10,11,12,13]. In a recent paper, Andrady et al. [13] compared the accelerated weathering of polyethylene in air and sea water. The authors found that the degradation occurs only in a very thin surface layer, and they, like other authors, attributed the retardation of the degradation with respect to the degradation in air to the lower availability of oxygen in sea water. In another paper, Gao et al. [12] attributed some protective role to the chlorine ion also. However, in this paper, the oxygen concentration in the tests with different chlorine ion concentrations is not reported. 

The degradation in water and sea water has also been investigated in oxo-degradable and biodegradable polymers [9,14]. In both papers, a low degradation of the biodegradable polymers was observed lower than that observed in air in composting environments. Furthermore, this behavior was attributed to the reduced availability of oxygen in the water. 

This work aims to investigate the effect of the environment and temperature on the photo-oxidation kinetic of polypropylene during accelerated weathering. Three different environments were investigated, namely, air, distilled water and sea water, at the temperatures of 40 and 70 °C. To our knowledge, the study of the effect of the temperature in the water has not been reported, and it is particularly interesting because increasing the temperature causes a relevant increase of the photo-oxidation kinetic to be observed. However, at the same time, increasing the temperature decreases the oxygen concentration in the water, and the lower availability of oxygen could reduce the photo-oxidation kinetic. The experimental results demonstrate that in our experimental conditions: (1) the degradation by photo-oxidation of the polypropylene is strongly retarded in the two water samples and (2) the increase of the temperature gives rise to a remarkable increase of the photo-oxidation kinetic, despite the lower availability of oxygen.

## 2. Materials and Methods

The polymer sample used in this work was a polypropylene copolymer Moplen RP34OH produced by LyondellBasell (LyondellBasell, Ferrara, Italy), with a melt flow index (MFI) of 1.8 g/10 min (230 °C/2.16 kg) and density 0.90 g/cm^3^.

The marine water was collected along the coast of Palermo in April 2022. In Table 1, the values of the dissolved oxygen and chlorine ion in the distilled and marine water measured at 25 °C are reported. 

Dissolved oxygen was measured using a Multi 3430 digital meter with an IDS FDO 925 digital dissolved oxygen sensor (WTW Inc., 11390 Amalgam Way, Gold River, CA 95670, USA). Chlorine ion was measured using electrochemical Ag sensors produced in the laboratory, using the linear scanning voltammetry (LSV) technique [15]. The environmental aging was carried out by keeping the samples of polypropylene in air, distilled water and marine water at the temperatures of 40 and 70 °C in a QUV under continuous irradiation. The lamps were UVB 313 nm with a UV irradiation peak at 313 nm. The samples were kept in aluminum trays immersed in water and covered by a film of PVC (a flexible, commercial film for food covering of about 10 μm thickness). The oxygen concentration remained almost constant during the test because the water was in equilibrium with the air above the water and the PVC film is permeable to the oxygen. In particular, the value of the Oxygen Transmission Rate (OTR) was more than 15,000 cm^3^/m^2^/24 h/atm, the limit of the instrument. The same arrangement was used for the samples irradiated in air. The trays were kept on the bottom of the QUV, under the lamps. The polypropylene did not undergo hydrolysis nor hydrothermal degradation. The degradation was, then, dependent only on the temperature and ultraviolet irradiation. Moreover, because the polymer was the same in all the tree environments, possible diffusion-limited oxygen (DLO) could not change the results relative to the three environments.

**Figure 1:**
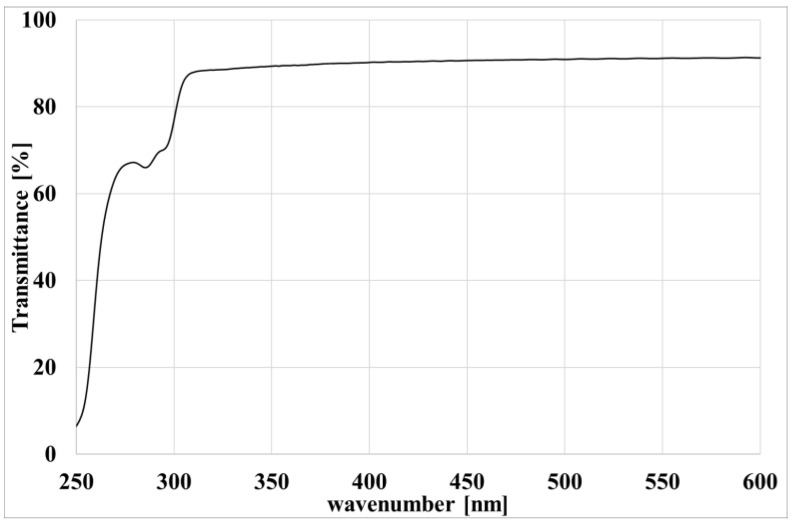
UV spectrum of the PVC films.

To avoid any possible effect on the UV transparency of the film due to the photo-oxidation, the films were changed every 24 h. The films were made of PVC with a very high transmittance in the UV wavelength range of the spectrum of the lamps (see Figure 1).

UV–vis absorption spectra of the samples were measured with a Specord 252 spectrometer (Analytik Jena, Jena, Germany) in the range 200–600 nm.

The rheological characterization was performed at 190 °C using an ARES G2 (TA Instruments, New Castle, DE, USA) plate–plate rotational rheometer in the frequency range 0.1 to 100 rad s^−1^.

FTIR spectra in ATR mode were obtained by using a Spectrum One spectrometer (Perkin-Elmer, Norwalk, CT, USA) equipped with integrated Spectrum One software. The spectra were obtained through 8 scans.

Mechanical properties, tensile strength (TS) and elongation at break (EB) were determined using an Instron (Instron, High Wycombe, UK) universal testing machine model 3365, at a crosshead speed of 100 mm min^−1^, whereas elastic modulus was measured at a speed of 1 mm min^−1^.

Thermal analysis was performed using a Setaram DSC131 Evo (Setaram Instrumentation 7 rue de l’Oratoire 69300 Caluire—France). The heating rate was 10 °C min^−1^ in a temperature range of 25 to 200 °C.

The Oxygen Transmission Rate (OTR) of the PVC film was evaluated through an Oxygen Permeation Analyzer model M8001 (Systech, llinois, Thame, UK), following the ASTM D3985 standard. OTR values were acquired at 23 °C with 0% RH by using high purity oxygen gas as the testing gas and high purity nitrogen gas as the carrier gas.

## 3. Results and Discussion

### 3.1. Photo-Oxidation Kinetic

**Figure 2:**
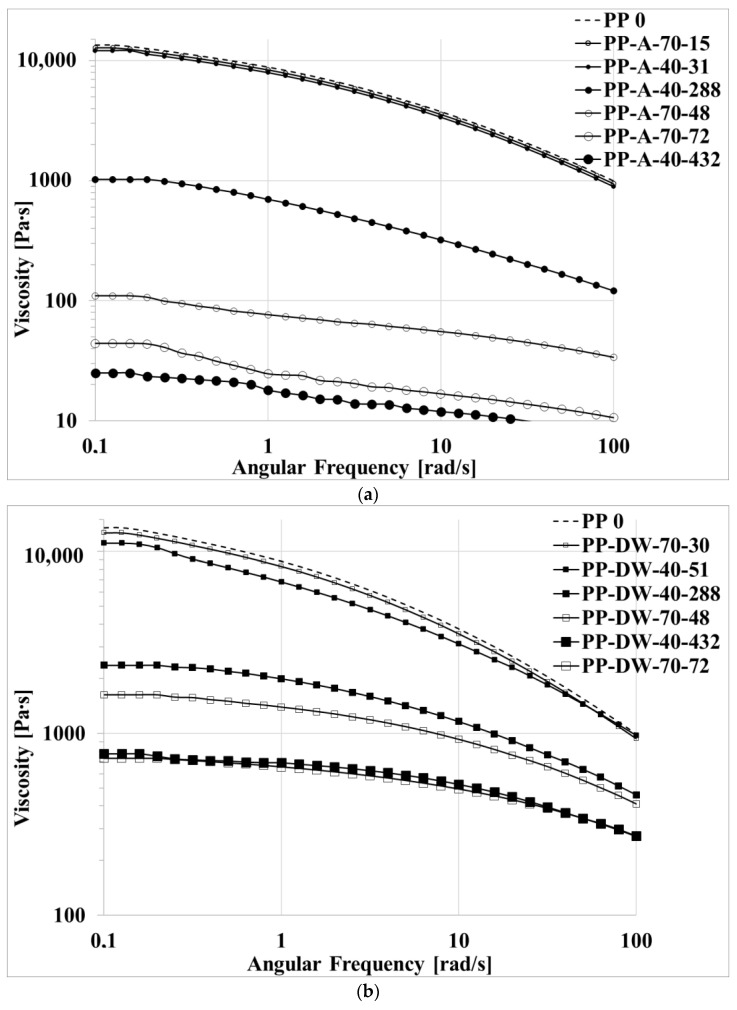

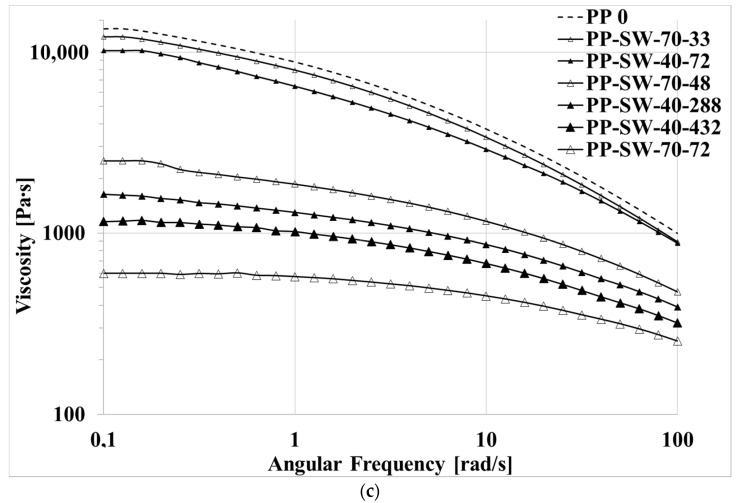
Flow curves of virgin PP and of photo-oxidized samples in (**a**) air, (**b**) distilled water and **(c)** sea water.

The flow curves of some selected samples photo-oxidized in the three different environments—in air, A; in distilled water, DW; in sea water, SW—and at the two temperatures, 40 and 70 °C, for given irradiation times are reported in Figure 2a–c. PP-A-70-15 means, for example, PP irradiated in air at 70 °C for 15 h.

**Figure 3:**
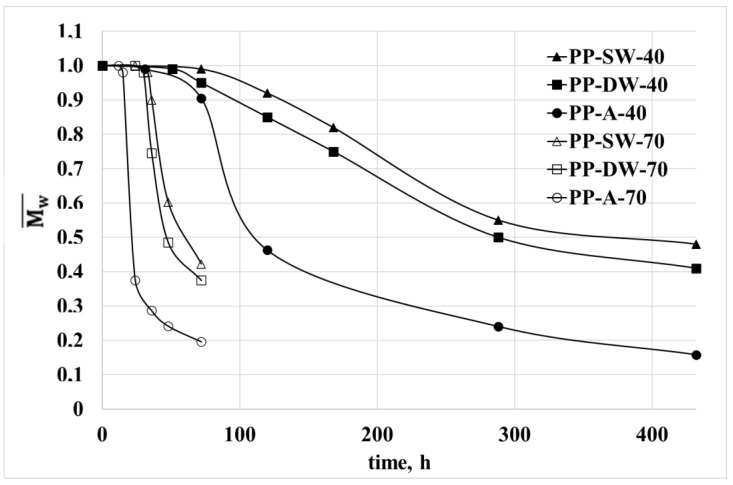
Dimensionless molecular weight as a function of the irradiation time in all the environmental conditions.

The viscosity of all the samples decreases with increasing irradiation times. Because the viscosity is strongly dependent on the molecular weight, this means that the PP sample undergoes a relevant degradation process. The decrease is much more pronounced when the temperature increases and when the photo-oxidation occurs in air. As for the samples photo-oxidized in the two water samples, the viscosity decreases slightly more in the distilled water. In Figure 3, the dimensionless molecular weight is reported for all the samples. The dimensionless molecular weight was calculated with the equation

η_0_ = kMw^^3.4^ (1)

where η_0_ is the Newtonian viscosity, K a constant and Mw the weight average molecular weight. Then, the dimensionless molecular weight, $\bar{M_{w}}$*,* of the sample at a given irradiation time (Figure 3) is

(2) Mw¯(t) = (η(t)/η0)^1/3.4

where η(t) is the Newtonian viscosity at a given irradiation time, t.

The molecular weight of the PP dramatically decreases with irradiation time and is, of course, a powerful index of the degradation of the polymer under UV irradiation. As previously stated, the decrease of the molecular weight and then the degradation of the polypropylene is very fast when the environment is air, and it is slower in the distilled water and the sea water. 

**Figure 4:**
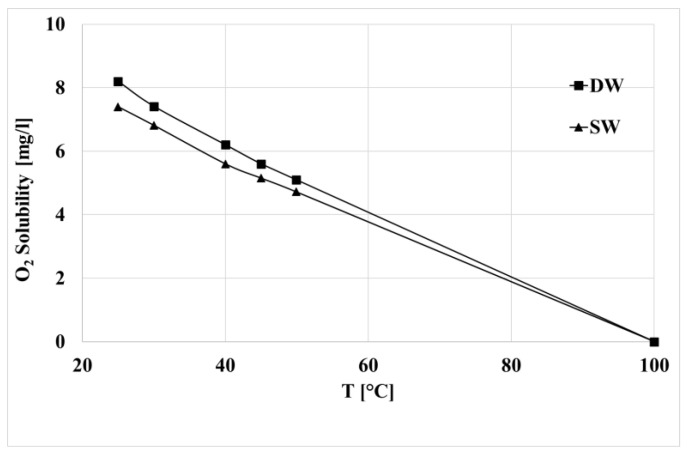
Concentration of oxygen as a function of the temperature for distilled and sea water.

The fast photo-oxidation in the air is, according to previous literature results, an expected result because the oxygen concentration in the air is undoubtedly higher than in both samples of water. In Figure 4, the values of the dissolved oxygen of the distilled and sea water are reported as a function of the temperature. It was possible to measure the two concentrations only up to 50 °C. The curves of the oxygen above these temperatures have been extrapolated by considering that at 100 °C, the dissolved oxygen in the water is zero.

Both at 40 and 70 °C, the concentration of oxygen in the distilled water is higher, but the difference is relatively small and decreases with the temperature. For this reason, the photo-oxidation kinetic is slower than in air, and the photo-oxidation kinetic in the two aqueous environments is slower in sea water. As for the effect of the temperatures, the photo-oxidation kinetic at the higher temperature is faster, although the concentration of oxygen is lower. From the dimensionless molecular weight curves, it is possible to evaluate the induction time and a kinetic constant because the chain scission is the first step of the photo-oxidation. The induction time was evaluated as the time at which the molecular weight decrease starts. The kinetic constant, K, was calculated as the slope at the beginning of the decrease of these curves. In Table 2, both values have been reported for all the environmental conditions.

The induction time is lower at higher temperature. Furthermore, at both temperatures, it is about one-half in the air with respect to the induction time in sea water. The values reported in Table 2 confirm that the photo-oxidation kinetic is higher with the increasing of the temperature and the concentration of oxygen. However, the induction time and kinetic constant in the two aqueous media approach one another at the higher temperature. This feature can be attributed to the fact that the difference between the concentrations of dissolved oxygen decreases with the increasing of the temperature. The induction times and the kinetic constants at 70 °C for the samples in water are very similar because the difference in the oxygen concentration is very low and lower than at 40 °C. 

### 3.2. FTIR–ATR Analysis

**Figure 5:**
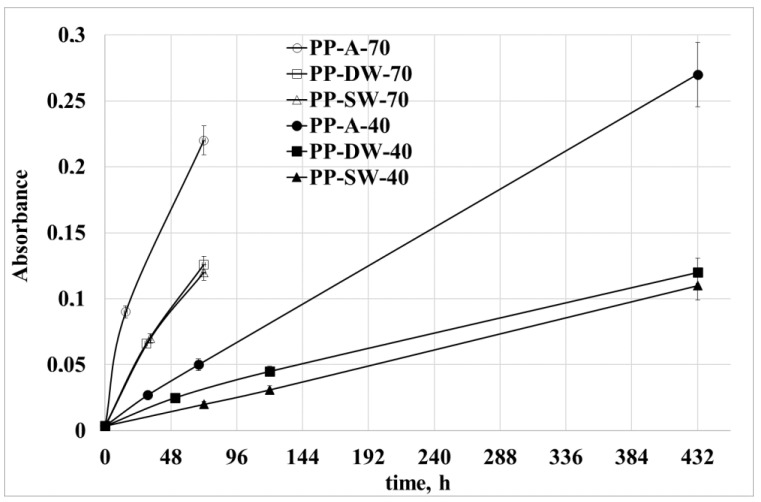
Peak height at 1720 cm^−1^ at 40 °C and 70 °C.

**Figure 6:**
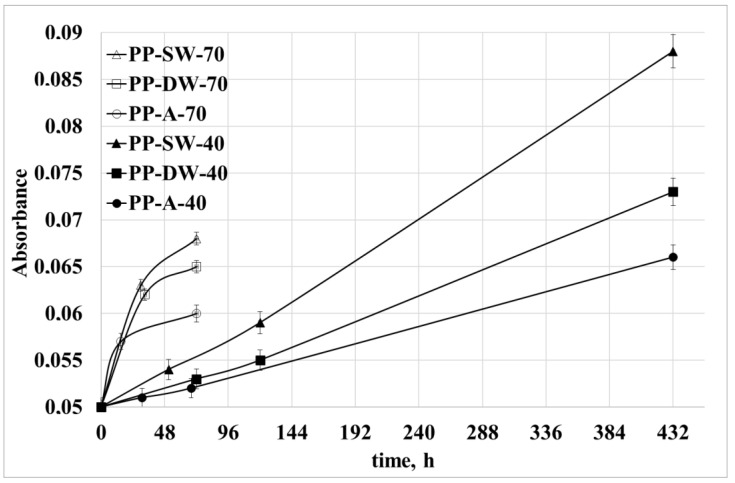
Peak height at 888 cm^−1^ at 40 °C and 70 °C.

The normalized absorbance at 1720 cm^−1^ (carbonyl index) is reported in Figure 5 for all the samples. In Figure 6, the normalized absorbance at 888 cm^−1^ (vinylidene index) is reported for all the samples.

The peak at 1720 cm^−1^ is representative of the C=O groups formed during oxidation. These groups increase with the increasing of the temperature and from sea water to distilled water and to the air. These last samples show the highest oxidation level due to the higher oxygen concentration. As for the vinylidene groups, the experimental data show the same trend with the temperature, but an opposite trend for the oxygen availability. Indeed, the largest growth is shown for the samples photo-oxidized in marine water, and the lowest growth is shown for the samples photo-oxidized in air. A possible interpretation of these data can be related to the fact that the lower availability of oxygen requires that a large number of radicals close with double bonds.

### 3.3. Mechanical Properties

**Figure 7:**
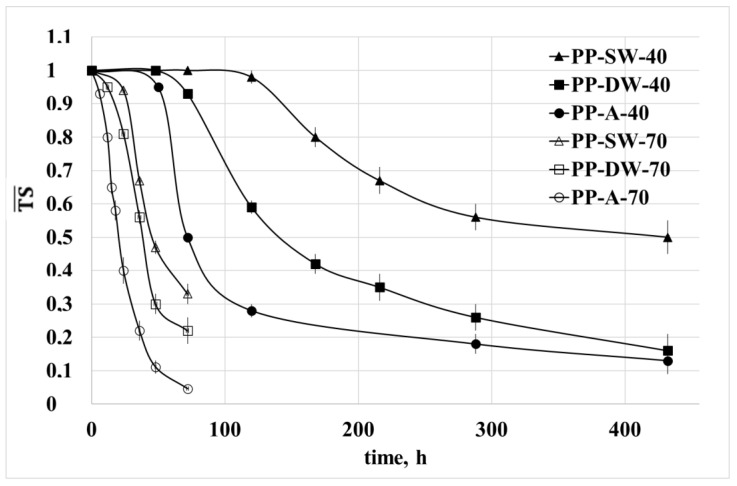
Dimensionless tensile strength as a function of the irradiation time for all the samples at the two temperatures.

**Figure 8:**
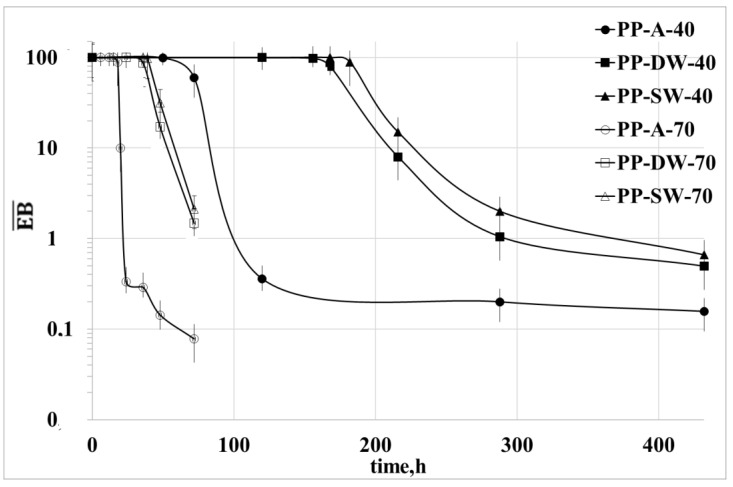
Dimensionless elongation at break as a function of the irradiation time for all the samples at the two temperatures.

The dimensionless values of the tensile strength, $\bar{TS}$, and elongation at break, $\bar{EB}$, are reported for all the investigated samples in Figure 7 and Figure 8 as a function of the irradiation time. The dimensionless values were calculated by dividing the values of these two properties by the values of the virgin PP.

The tensile strength and, in particular, the elongation at break are mechanical properties strongly dependent on the morphology and molecular structure of the polymers. The decrease of TS and EB, in particular, suggests a rapid change of morphology and molecular structure induced by the photo-oxidation [16,17]. For both temperatures, the decrease of both properties is very fast for the sample exposed in air and at higher temperature, while it is lower for the two samples exposed in water and at the lower temperature. Moreover, the change with the irradiation time of TS and EB of the 2 samples exposed in water at 70 °C is almost the same, while some difference is observed for the same samples exposed at 40 °C. This evidence confirms that the degradation in the 2 aqueous media is very similar due to the similar oxygen content at 70 °C. 

## 4. Conclusions

In this work, the photo-oxidation of a polypropylene sample in three different environments—air, distilled water and sea water—and at two different temperatures was assessed by means of rheological, mechanical and spectroscopic measurements. All the results suggest that, as expected, the photo-oxidation kinetic is higher and the induction time lower when the sample irradiates in air. In the water, and in particular in the sea water, the photo-oxidation kinetic is slower and the induction time higher. The molecular weight and the ultimate mechanical properties decrease and the carbonyl groups increase with the increasing of the irradiation time and the temperature. With respect to the degradation phenomena, the main difference among the three different environments is the different concentration of oxygen. For this reason, the degradation is very fast in the air, while it is slower in water. In particular, the photo-oxidation is slightly faster in distilled water, where the oxygen concentration is slightly higher. Furthermore, the photo-oxidation kinetic increases with the temperature also in water, although the oxygen concentration in water decreases. Finally, the photo-oxidation kinetics at higher temperature in the two types of water approach one another because the difference in the oxygen concentration decreases with the increasing of the temperature.

## Figures and Tables

**Table 1:** Oxygen and chlorine ion in distilled and marine water.

Water	Oxygen (mg L^−1^)	Chlorine Ion (g L^−1^)
Distilled water	8.15	0
Marine water	6.95	21.1

**Table 2:** Induction times and kinetic constants for the photo-oxidation of the polypropylene in all the environmental conditions.

	K, t^−1^	Induction Time, h
PP–A-40	0.0011	31
PP-DW-40	0.00088	51
PP-SW-40	0.00066	72
PP–A-70	0.021	15
PP-DW-70	0.009	30
PP-SW-70	0.0085	33
